# Characterization of aberrant glycosylation associated with osteoarthritis based on integrated glycomics methods

**DOI:** 10.1186/s13075-023-03084-w

**Published:** 2023-06-12

**Authors:** Hanjie Yu, Mingxiu Li, Jian Shu, Liuyi Dang, Xin Wu, Yuzi Wang, Xuan Wang, Xin Chang, Xiaojuan Bao, Bojing Zhu, Xiameng Ren, Wentian Chen, Yi Li

**Affiliations:** 1 Laboratory for Functional Glycomics, College of Life Sciences, Northwest University, Xi’an, China; 2 Department of Foot and Ankle Surgery, Honghui Hospital, Xi’an Jiaotong University, 76 Nanguo Road, Xi’an, 710054 Shaanxi Province China; 3 The Second Clinical Medical College of Shaanxi University of Chinese Medicine, Xianyang, China; 4 College of Life Science, Northwest University, Xi’an, China

**Keywords:** Osteoarthritis, Glycosylation, Lectin microarray, LC–MS, Intact glycopeptides, Site-specific glycosylation

## Abstract

**Background:**

Osteoarthritis (OA) is the most common form of arthritis, affecting millions of aging people. Investigation of abnormal glycosylation is essential for the understanding of pathological mechanisms of OA.

**Methods:**

The total protein was isolated from OA (*n* = 13) and control (*n* = 11) cartilages. Subsequently, glycosylation alterations of glycoproteins in OA cartilage were investigated by lectin microarrays and intact glycopeptides analysis. Finally, the expression of glycosyltransferases involved in the synthesis of altered glycosylation was assessed by qPCR and GEO database.

**Results:**

Our findings revealed that several glycopatterns, such as α-1,3/6 fucosylation and high-mannose type of N-glycans were altered in OA cartilages. Notably, over 27% of identified glycopeptides (109 glycopeptides derived from 47 glycoproteins mainly located in the extracellular region) disappeared or decreased in OA cartilages, which is related to the cartilage matrix degradation. Interestingly, the microheterogeneity of N-glycans on fibronectin and aggrecan core protein was observed in OA cartilage. Our results combined with GEO data indicated that the pro-inflammatory cytokines altered the expression of glycosyltransferases (ALG3, ALG5, MGAT4C, and MGAT5) which may contribute to the alterations in glycosylation.

**Conclusion:**

Our study revealed the abnormal glycopatterns and heterogeneities of site-specific glycosylation associated with OA. To our knowledge, it is the first time that the heterogeneity of site-specific N-glycans was reported in OA cartilage. The results of gene expression analysis suggested that the expression of glycosyltransferases was impacted by pro-inflammatory cytokines, which may facilitate the degradation of protein and accelerate the process of OA. Our findings provide valuable information for the understanding of molecular mechanisms in the pathogenesis of OA.

**Supplementary Information:**

The online version contains supplementary material available at 10.1186/s13075-023-03084-w.

## Background

Osteoarthritis (OA) is the most common form of arthritis, affecting 7% of the global population [1]. As a degenerative joint disease, OA is characterized by the loss of extracellular matrix (ECM) proteins, subchondral bone remodeling, and articular cartilage deterioration [2–4]. The degradation of cartilage is not only the hallmark of OA but also caused an inflammatory process and release of inflammatory cytokines [5, 6]. These cytokines promote the expression of matrix metalloproteinases (MMPs) and aggrecanases and block the synthesis of ECM by suppressing the expression of ECM structural proteins [7–9]. As a result, it accelerated the degradation of ECM molecules and breakdown the balance of synthesis and degradation in cartilage [10]. Apart from that, numerous factors (including synovium, ligaments, and muscles) are also involved in the pathogenesis of OA, and the pathological mechanism of OA remains needs to be illustrated.

Glycosylation is the most common and complex form of post-translational modification of proteins. The glycans are not only indispensable for the correctly folded and mature conformation of proteins but are also involved in diverse biological processes, such as cell recognition, signal transduction, and inflammation [11, 12]. It is known that aberrant glycosylation is correlated with diseases, such as cancers [13]. Recent studies reported that the glycosylation of cartilage proteins was altered during the development of OA, for instance, the alterations in sialylated and fucosylated N-glycans of cartilage were observed preceding the histological change of OA cartilage [14]. The heterogeneity is a characteristic of glycans, which poses an obstacle to the identification of site-specific glycosylation [15]. Recently, the glycan structures and their attached glycoproteins can be identified and quantified simultaneously by using mass spectrometry-based intact glycopeptides analysis [16, 17]. However, the precise information about the alterations in glycosylation of cartilage proteins and the roles of abnormal glycosylation in the process of OA is still not fully understood.

In this study, the alterations in glycopatterns related to OA were accessed by lectin microarrays. Meanwhile, the heterogeneities of site-specific glycosylation associated with OA were further investigated by intact glycopeptides analysis. The expression of glycosyltransferases involved in the altered glycoforms of OA cartilage and OA cell model were evaluated by GEO data and qPCR. Our study took insights into alterations in glycosylation of cartilage and provided more information to understand the pathological mechanism of OA.

## Materials and methods

### Collection of knee cartilage tissue specimens

Cartilage tissues from knee joints were obtained from operations performed from 2021 to 2022 in the Department of Bone Microsurgery, Foot and Ankle Surgery, and Joint Surgery of Xi’an Honghui Hospital. The collection was approved by the Ethical Committee of Xi’an Honghui Hospital (XJTUAE2023-1346). Written informed consent was received from participants. The study was conducted under the ethical guidelines of the Declaration of Helsinki. The inclusion criteria were as follows: the patients were diagnosed as primary knee joint OA, and Kellgren-Lawrence grading of knee X-ray ≥  iii. Patients with rheumatoid arthritis, infectious arthritis, traumatic arthritis, and other immune system diseases were excluded. As a result, 13 patients with knee OA were enrolled in the OA group and 11 tibia platform and distal femur comminuted fracture patients were recruited into the control group. The information of participants was summarized in Table S1.

### Histopathological evaluation of OA

To evaluate the degree of degradative changes in the cartilage, cartilaginous tissue from OA patients and healthy volunteers were assessed by hematoxylin–eosin (H&E) staining. In brief, cartilages were embedded in paraffin and sectioned at 6 μm. After deparaffinization and hydration, the sections were stained with H&E (TEASEN Biotechnology, China) according to the manufacturer’s instructions.

### Cartilage protein extraction

The total protein was extracted by using T-PER Tissue Protein Extraction Reagent (Thermo, USA). Briefly, the cartilaginous tissue was homogenized by 1 mL of T-PER reagent with 1% (*v/v*) of protease inhibitor cocktail (MedChemExpress, Shanghai, China), and sonicated for 5 min under an ice bath. The samples were centrifuged at 12,000 × g for 15 min at 4℃, and the supernatant was transferred into a new tube. The concentration of total proteins was determined using a BCA assay (Beyotime Biotechnology, Nantong, China).

### Lectin microarray and data analysis

The manufacture of the lectin microarrays and data analysis were performed as described previously [18–20]. Briefly, 100 μg of proteins were labeled with Sulfo-Cy3 NHS ester potassium salt (J&K Scientific) and purified using a Sephadex G25 Desalting column (PD-10, GE Healthcare). The lectin microarrays were blocked with blocking buffer (containing 2% (*w/v*) BSA and 0.1% Tween-20 in PBS, pH 7.4) for 1 h. Then, 4 μg of labeled proteins were incubated with lectin microarray at 37 ℃ for 3 h. After washing, the microarrays were scanned using a confocal scanner (4100A, AXON Instruments, USA), and the fluorescence intensities of microarrays were extracted by GenePix 7.0 software (Axon). After filtration and normalization, the parallel datasets were compared with each other based on fold changes according to the following criteria: fold changes ≥ 1.5 or ≤ 0.67 and *p* < 0.05 indicated upregulation or downregulation, respectively. Significant differences between groups were evaluated using the Mann–Whitney test. Detailed information about microarray data analysis was summarized in Supplementary Materials. The normalized fluorescence intensities (NFIs) of lectins were further analyzed by hierarchical cluster analysis (HCA). Principal component analysis (PCA) was performed by using bioinformatics (an online platform for data analysis and visualization, https://www.bioinformatics.com.cn).

### Lectin and immune-blotting

Firstly, 30 μg of cartilage proteins from OA (*n* = 3) and control subjects (*n* = 3) were separated by 3% stacking gel and 10% polyacrylamide resolving gel. Then, the protein bands in gels were transferred into PVDF membranes (0.22 μm Millipore, Bedford, MA, USA) and blocked by Carbo-free blocking solution (vector labs, Burlingame, CA) for 1 h at room temperature. After that, the membranes were incubated with 20 μg/mL of Cy5-labeled *Wheat Germ Agglutinin* (WGA), *Sophora Japonica Agglutinin* (SJA), and *Canavalia ensiformis Agglutinin* (ConA) respectively. After washing with TBST buffer, the image was acquired by a STROM fluorImager (Molecular Dynamics, Sunnyvale, CA, USA). The gray value of the protein bands was extracted by ImageJ software(NIH). The expression level of GAPDH served as control.

### Lectin histochemistry

Lectin histochemistry was performed as previously described [21]. After deparaffinization and hydration, the sections were blocked and incubated with Carbo- 20 μg/mL Cy3-labeled PSA at 4 ℃ overnight. The nucleus was stained with 4,6-Diamidino-2-phenylindole (DAPI, 1 µg/mL in PBS) (Roche; Basel, Switzerland). Finally, sections were scanned by Laser Scanning Confocal Microscope FV 1000 (Olympus, Tokyo, JPN).

### Protein digestion

Briefly, 1 mg of pooled proteins from OA (*n* = 13) and control groups (*n* = 11) were denatured in 8 M urea/1 M NH_4_HCO_3_ buffer, respectively. Then, the proteins were reduced by 5 mM dithiothreitol (DTT) at 37 °C for 1 h and alkylated by 15 mM iodoacetamide (IAM) at room temperature in the dark for 30 min. After diluting two times with deionized water, the proteins were digested with sequencing grade trypsin (Promega, USA, WI; proteins: enzyme, 100:1, w/w) for 2 h with shaking at 37 °C. The proteins were further digested for the second time with the same amount of trypsin (proteins: enzyme, 100:1, w/w) overnight at 37 °C with shaking after diluting another four times. The pH of the solution was adjusted to < 2 with 10% TFA. The samples were centrifuged at 15,000 g for 10 min to remove any particulate matter and the peptides in supernatants were desalted by the C18 columns (Waters, USA). Finally, the peptide concentrations were measured by BCA reagent (Beyotime Biotechnology, Nantong, China).

### Enrichment of glycopeptides

The intact glycopeptides were enriched using an anion exchange reversed phase (MAX) column. Firstly, the Oasis MAX cartridge (Waters, USA) was equilibrated with ACN, 100 mM triethylammonium acetate (Sigma), ddH_2_O, and 95% ACN/1% TFA. Then, the desalted peptides were loaded onto cartridges twice after being dried and reconstituted in 95% ACN/1% TFA. The cartridges were washed with 95% ACN/1% TFA three times. The glycopeptides were eluted with 600 μl of 50% ACN/0.1% formic acid (FA, Sigma). Finally, the glycopeptides were dried and resuspended in 20 μL of 0.1% FA. One microgram of glycopeptides was applied to LC–MS/MS analysis.

### LC–MS/MS

One microgram of glycopeptides was separated on an EASY-nLC™ 1200 instrument (Thermo Fisher Scientific, Germany) with the use of Acclaim PepMap100 precolumn (2 cm, 75 µm i.d., 3 µm) and Acclaim PepMap100 separating column (50 cm, 75 µm i.d., 3 µm) operating at 300 nL/min. The mobile phase A (0.1% FA) and mobile phase B (0.1% FA / 80% ACN) with a flow rate of 200 nL/min. The parameters used for intact glycopeptides analysis were the same as previously described [22, 23].

### Intact glycopeptides identification and label-free quantification

The intact glycopeptide MS data were searched by Byonic (Protein Metrics Inc) using the human *N*-glycan database with no multiple fucoses and the human reference proteome protein database (www.uniprot.org, downloaded in Aug 2018). The search parameters were set as follows: cysteine carbamidomethylation (C, + 57.0215 Da) was set as a fixed modification; methionine oxidation, protein N-terminal acetylation (+ 21.011 Da), and oxidization (M, + 15.9949 Da) were set as variable modifications; up to two missed cleavage were allowed for trypsin digestion; 10 ppm and 0.02 Da mass tolerance was set for precursor and MS/MS ions, respectively. The glycopeptides with Byonic Score > 300 were selected to further analysis. The quantification of intact glycopeptides was performed by Proteome Discoverer v2.3 (Thermo Fisher Scientific) with a label-free quantification (LFQ) approach. The significantly changed intact glycopeptides were screened with a threshold of ratios ≥ 2 or ≤ 0.5 between OA and control groups and *p*-value < 0.05.

### Bioinformatics analysis

The Protein–Protein Interaction Networks Functional Enrichment Analysis was performed using STRING (Version 11.5) [24]. Gene Ontology (GO) enrichment analysis and Kyoto Encyclopedia of Genes and Genomes (KEGG) pathway analysis of the differential glycoproteins were performed by Database for Annotation, Visualization, and Integrated Discovery (DAVID) online software. The thresholds of FDR < 0.05, fold changes were > 2 or < 0.5, and count ≥ 3 were applied as filters on functional analysis. The expression of glycosyltransferases involved in the synthesis of N-glycan was re-analyzed using Gene Expression Omnibus (GEO) data set (GSE51588) [25]. The Volcano Plots were generated by GEO2R online software in NCBI.

### Chondrocyte culture and treatment

The human chondrocyte cell line C28/I2 (C28) was cultured in DMEM/F12 supplemented with 10% FBS (NEWZERUM, New Zealand), 100 U/ml penicillin, and 100 μg/mL streptomycin (MCE) in a humidified incubator at 37 °C in the presence of 5% CO_2_. Before treatment, chondrocytes were serum-starved for 12 h and then stimulated with recombinant human interleukin 1β (IL-1β, Novoprotein, China) at a concentration of 10 ng/mL for 48 h. The unstimulated chondrocytes were used as controls synchronously. After stimulation, the cells were washed with sterile PBS and collected.

### Senescent cell staining

The senescent cells were stained using a senescence β-galactosidase staining kit (Beyotime). Briefly, the IL-1β treated or untreated C28 chondrocytes were fixed with 4% paraformaldehyde (Solarbio, Beijing, China) and stained with 1 mL of staining solution at 37 ℃ overnight. The SA-β-gal positive chondrocytes were observed using bright-field microscopy.

### Isolation of RNA and semi-qPCR

Total RNA from chondrocytes and cartilage tissue was extracted by Trizol Reagent (Life Technologies, Carlsbad, CA) according to the manufacturer’s protocol. Then, 1 μg of total RNA was reverse-transcribed using Evo M-MLV RT Kit (Accrate Biotechnology, Hunan, China), and qPCR was performed using a ViiA 7 Real-Time PCR System (Applied Biosystems, USA). SYBR Green-based three-step RT–qPCR was performed using SYBR® Green Premix Pro Taq HS qPCR Kit (Accrate Biotechnology). The primer sequences were retrieved from the online PrimerBank database (https://pga.mgh.harvard.edu/primerbank/index.html). The information of primers was summarized in Table S2.

## Results

### Assessment of abnormal glycopatterns in cartilage from OA patients

**Fig. 1:**
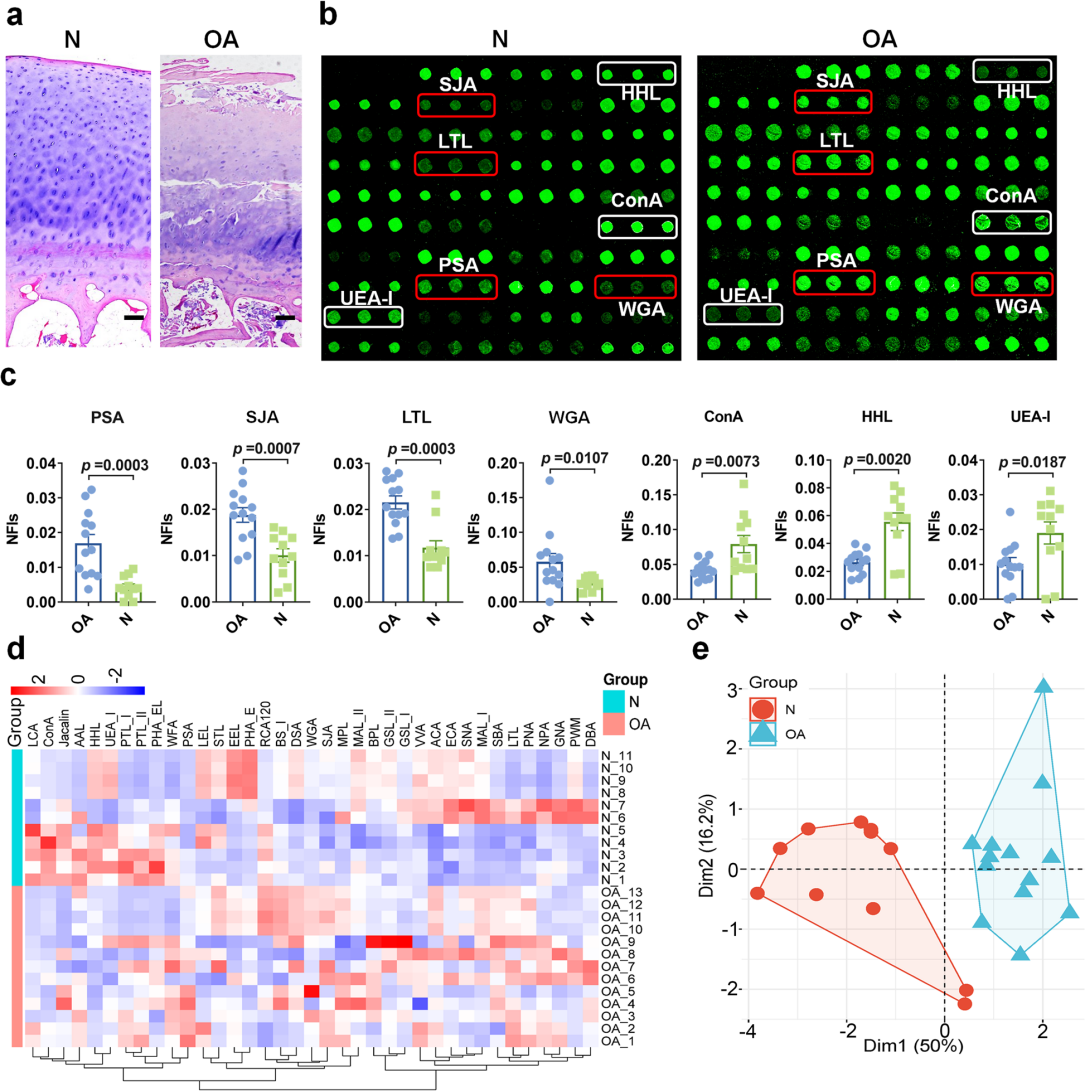
The glycopatterns exhibited significant differences between normal and OA cartilage. **a** Representative H&E staining images of OA and normal articular cartilages. The articular cartilage sections from OA patients displayed typical characteristics of OA compared with normal cartilage. Scale bar = 100 μm. **b** The representative scanned images of OA and normal cartilage. The lectins with increased or decreased NFIs in OA cartilage are marked with red or white boxes respectively. **c** The NIFs of 7 lectins showed significantly altered in OA (*n* = 13) to control (*n* = 11) cartilage based upon foldchange and *p* value. The data were represented as Mean with SEM. **d** Hierarchical clustering analysis of the 37 lectins with OA and control groups. Glycan profiles of normal and OA cartilage were clustered (average linkage, correlation similarity). Samples are listed in rows and the lectins are listed in columns. The color and intensity of each square indicated relative fluorescence intensities of lectins in the row. Red, high; blue, low; white, medium. **e** Principal component analysis of the difference between normal and OA cartilages. The NFIs of 7 lectins from OA and control groups were subjected to principal component analysis, normal and OA samples were indicated by cyan triangle and red dot, respectively

The H&E staining results revealed that knee sections from OA patients displayed several common pathologic changes, including damaged cartilage surface and nonuniform distribution of chondrocytes (Fig. 1a). As shown in Fig. 1b, the fluorescence intensities of several lectins (such as PSA and SJA) were altered in OA cartilages compared to normal cartilages (controls). Notably, the glycopatterns of Fucα-1,3/6GlcNAc (bound by lectins: PSA, LTL), glycans terminal in GalNAc and Gal (bound by lectins: SJA), as well as multivalent sialic acids, and (GlcNAc)n were significantly increased, however, high mannose type of N-glycan (bound by lectins: ConA, HHL) and Fucα1-2Galβ1-4Glc(NAc) (bound by lectins: UEA-I) were significantly decreased in OA cartilages compared to controls (Fig. 1c and Table S3). The result of HCA revealed that the OA and control samples were clustered into different categories by the NFIs of lectins (Fig. 1d). Furthermore, the result of PCA revealed that OA and controls located in different areas without overlapping, which indicated that the glycopatterns identified by these 7 lectins (such as PSA, WGA, and LTL) were different between OA and normal cartilages (Fig. 1e). These results indicated the alteration in glycopatterns of cartilage are characters of OA.

### Validation of abnormal Glycopatterns associated with OA

**Fig. 2:**
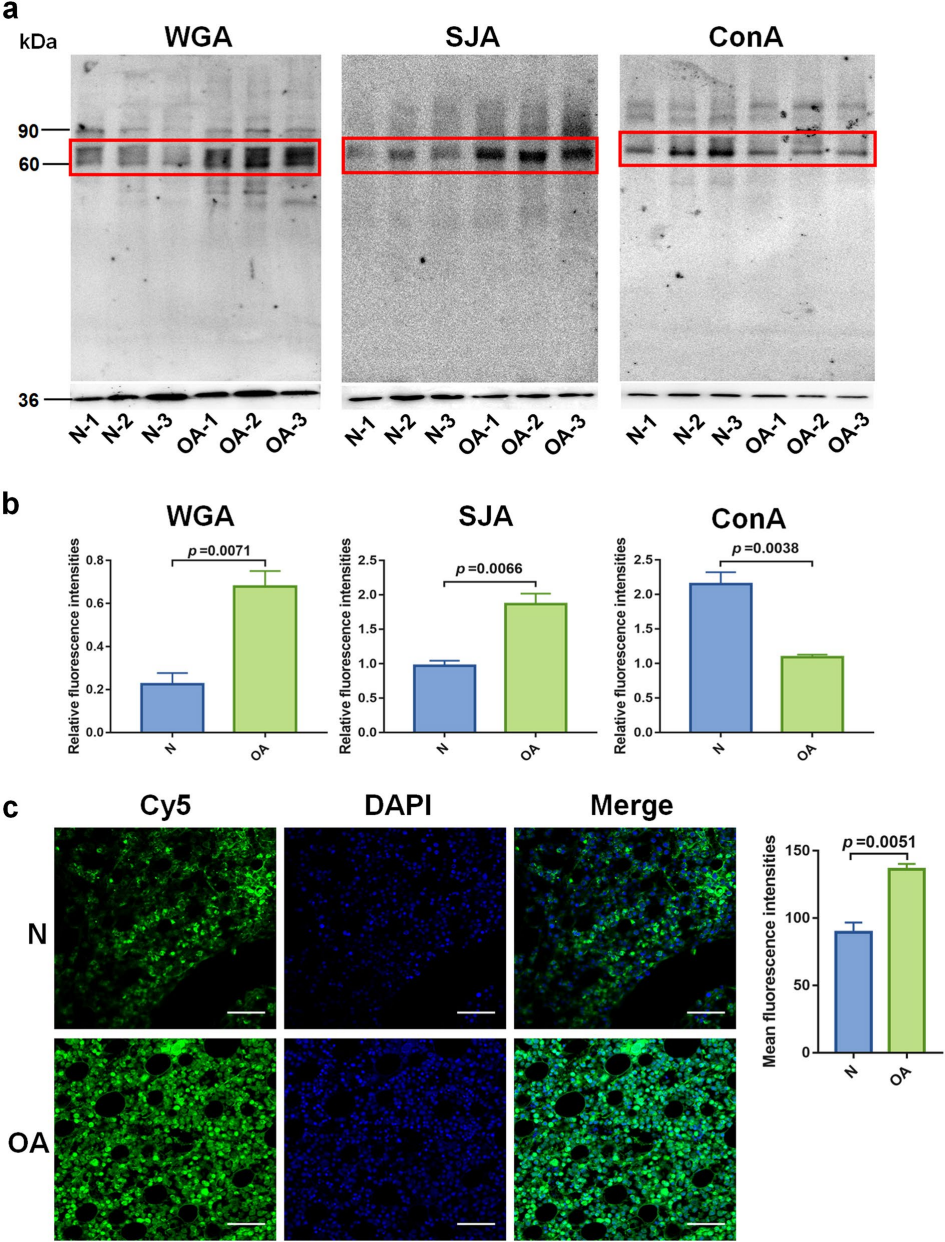
Validation of the altered glycopatterns in OA cartilages. **a** Binding patterns of glycoproteins in cartilage proteins from OA patients (*n* = 3) and controls (*n* = 3) for WGA, SJA, and ConA staining. The protein bands showed differences between OA and control cartilages marked with red frames. The expression level of GAPDH served as the loading control. **b** The gray value of the different protein bands was measured by ImageJ software, and the gray values of the selected bands were represented as mean with SEM. **c** The results of lectin histochemistry. The articular cartilage sections from healthy and OA patients were stained with Cy5-labeled PSA. The images were acquired at the same conditions. Quantification was shown to the right. Data were obtained from OA (*n* = 3) and control (*n* = 3) cartilage sections. Mean fluorescence intensities were represented as mean with SEM. Scale bar = 100 μm

Firstly, lectin blotting was used to confirm the alterations in glycopatterns associated with OA. WGA and SJA blotting results revealed that the staining intensities of the protein band about 60 kDa in OA cartilages were stronger than controls. Conversely, ConA showed a weaker binding to protein band about 60 kDa in OA cartilages than controls (Fig. 2a, b). The results of lectin histochemistry indicated that an increased level of α-1,6 fucosylation was observed in OA cartilage sections compared to controls. In addition, the staining area of PSA was mainly located in the cytoplasm and cytomembrane (Fig. 2c). Collectively, these results demonstrated that the alterations in glycopatterns are characteristics of OA cartilage.

### Identification of intact glycopeptides in OA and control cartilage

**Fig. 3:**
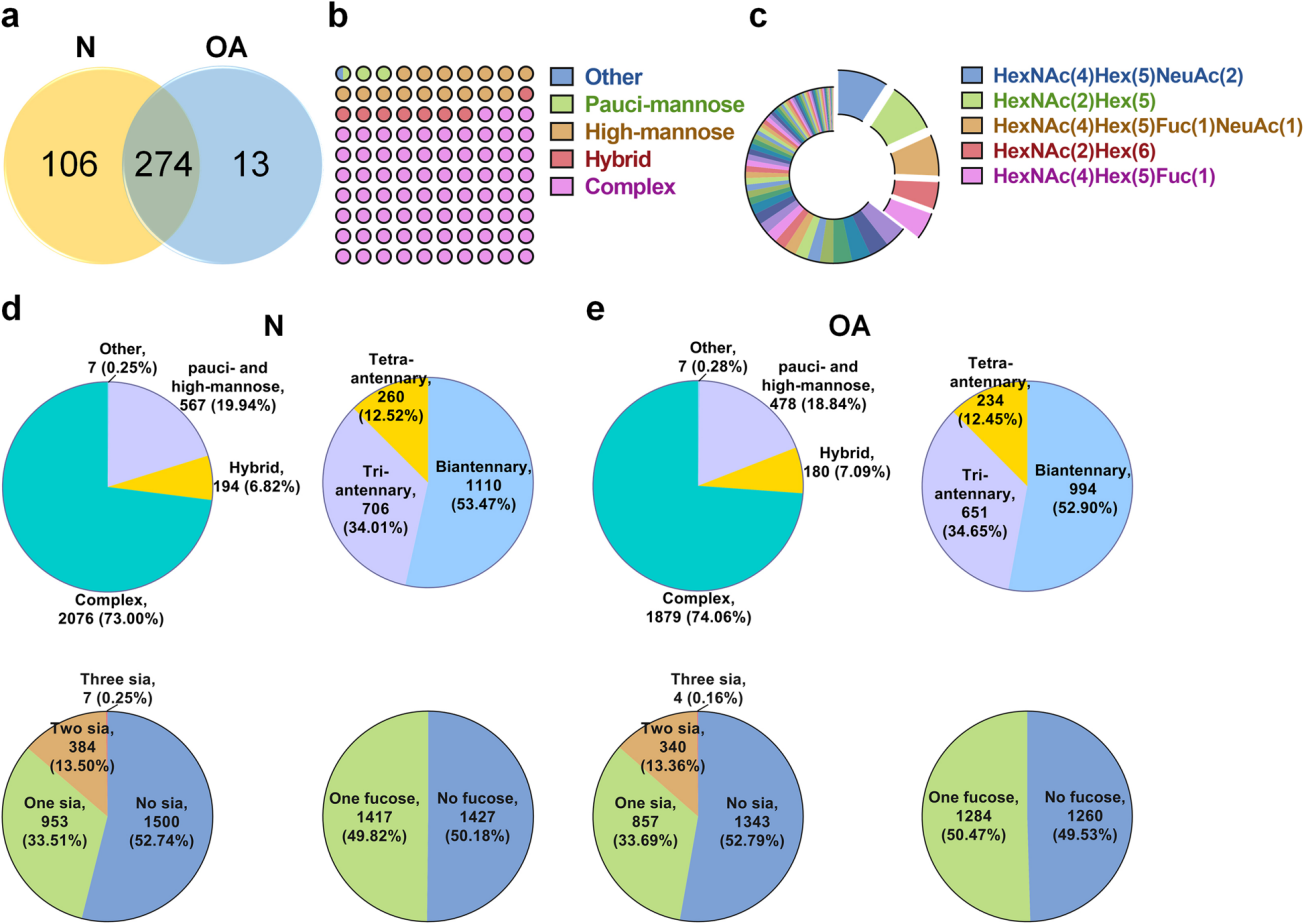
Characterization of intact glycopeptides from OA and control cartilages. **a** The number of intact glycopeptides identified in the control and OA cartilages. **b** The percentages of different types of N-glycans identified in glycopeptides. **c** The PSMs percentages of different glycan compositions identified in OA and control cartilages. The top five glycan compositions were labeled. **d**, **e** Distribution of glycan subtypes, numbers of antennary, sialic acid, and fucose modifications from intact glycopeptides identified from control (**d**) and OA (**e**) cartilages

Totally, 393 intact glycopeptides from 74 glycoproteins were identified, which consists of 109 glycosites and 85 different glycan compositions. Among these, 13 and 106 unique intact glycopeptides were identified from OA and control cartilages (Fig. 3a). The detailed information about glycopeptides identified in OA and control cartilages was summarized in Table S4. Among 85 glycan compositions, most of them were complex type *N*-glycans (72.77%), with a small portion of hybrid type of N-glycans (8.14%), pauci-mannose (2.54%) and high-mannose type (16.03%) and (Fig. 3b). The top five glycan compositions were complex type N-glycans (N4H5S2, N4H5F1S1, N4H5F1, and N5H5S1) and high mannose glycans (N2H5 and N2H6), which took up 35.48% PSMs of all glycan compositions (Fig. 3c). Up to 31 N-glycan compositions were identified at N333 (TVYVHAN^333^QTGYPDPSSR) of aggrecan core protein (ACAN), which is a major component of the extracellular matrix of cartilaginous tissues (Table S4). By calculating the PSMs of intact glycopeptides, it revealed that more than 70% of glycosites were occupied by complex glycans (2076 PSMs (73.00%) in control, and 1879 PSMs (74.06%) in OA). The bi- and tri-antennary N-glycans were the majority type of complex types of N-glycans in both normal and OA cartilage. However, the number of fucosylated N-glycans and sialylated glycans showed no significant differences between OA and control cartilages (Fig. 3d, e).

### Differentially expressed intact N-glycopeptides in OA cartilage

**Fig. 4:**
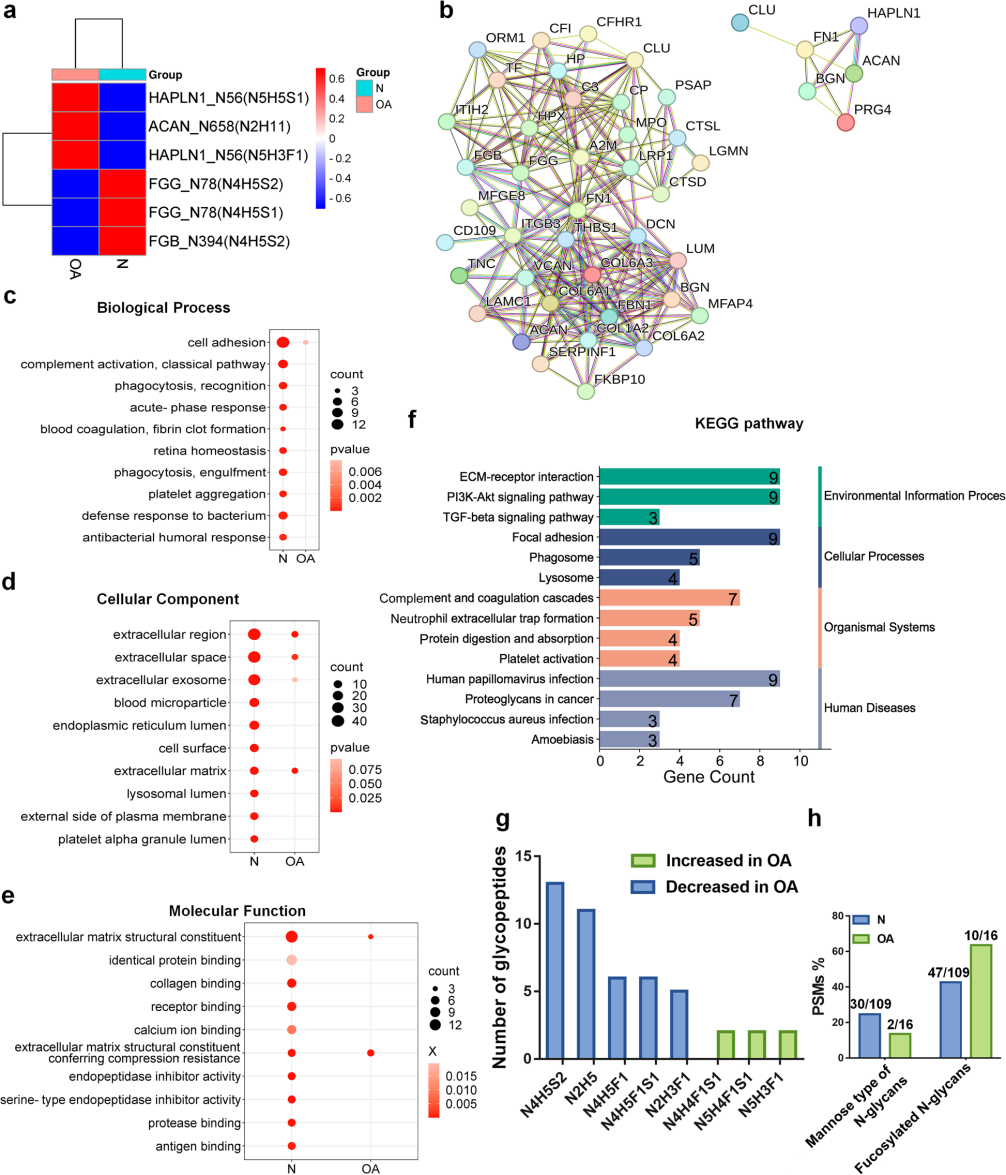
Comparison analysis of the altered glycopeptides in OA cartilage. **a** Profiling of three increased glycopeptides and three decreased glycopeptides in OA cartilages. **b** Protein–protein interaction network analysis of the significantly decreased (left) or increased (right) glycoproteins in OA cartilage. **c** Biological process, **d** cellular component, **e** molecular function, and **f** KEGG pathway analysis of significantly differential glycoproteins in OA cartilage. **g** Frequencies of altered site-specific glycans in OA cartilage. **h** The PSMs of glycopeptides modified with pauci- and high-mannose type of N-glycans and fucosylated N-glycans in the differential glycopeptides

With a criterion of fold change (FC) > 2 or < 0.5 and *p* < 0.05, over 97% of intact glycopeptides remained stable between OA and control cartilages. Six intact glycopeptides, including GGN^56^VTLPCK_N5H5S1, GGN^56^VTLPCK_ N5H3F1, and TVYLYPN^658^QTGLPDPLSR_N2H11 were increased, while, VDKDLQSLEDILHQVEN^78^K_N4H5S1, VDKDLQSLEDILHQVEN^78^K_N4H5S2, and GTAGNALMDGASQLMGEN^394^R_ N4H5S2 were decreased in OA compared to control cartilages (Fig. 4a). In addition, 106 and 13 intact glycopeptides from 47 and 6 glycoproteins were uniquely detected in control and OA cartilage respectively. The differentially expressed glycopeptides were summarized in Table S5. The glycoproteins mapped by these differentially expressed glycopeptides were subjected to further bioinformatics analysis. Two close interactions were observed among the glycoproteins that decreased in OA cartilage, which were mainly located in the ECM and extracellular region and involved in the ECM-receptor interaction and activation of the complement system. In contrast, the glycoproteins that increased in OA cartilage were mainly located in collagen-containing extracellular matrix and involved in glycosaminoglycan binding (Fig. 4b). GO analysis revealed that glycoproteins decreased in OA cartilages were mainly involved in cell adhesion, complement activation, classical pathway (Fig. 4c). These proteins were mainly localized in the extracellular region and extracellular space (Fig. 4d) and the molecular function of these proteins was primarily extracellular matrix structural constituent, identical protein and collagen binding (Fig. 4e). KEGG pathway analysis revealed that glycoproteins decreased in OA cartilages were involved in ECM-receptor interaction, PI3K-Akt signaling pathway and focal adhesion (Fig. 4f).

Next, we classified the glycan structures on the differentially expressed intact glycopeptides, and found that N4H5S2, N2H5, N4H5F1, N4H5F1S1, and N2H3F1 mostly decreased in OA cartilages, while the fucosylation glycans (N4H4F1S1, N5H4F1S1, and N5H3F1) were often increased in OA cartilages (Fig. 4g). Although the total percentages of pauci- and high-mannose N-glycan and fucosylated N-glycans did not show significant differences between OA and control cartilages, we found that 30 out of 109 and 47 out of 109 glycopeptides that decreased in OA cartilages were modified with pauci- and high- mannose N-glycans (92 PSMs, 24.66%) and fucosylated N-glycans (159 PSMs, 42.63%), respectively. By contrast, 2 out of 16 and 10 out of 16 N-glycopeptides increased in OA cartilages were modified with high-mannose N-glycans (6 PSMs, 13.64%) and fucosylated N-glycans (28 PSMs, 63.64%) (Fig. 4h).

### Microheterogeneity of site-specific N-glycans in cartilaginous proteins

Since the differences in site-specific N-glycopeptides were identified in OA and control cartilages, we further compared the abundance of N-glycopeptides at the glycosite level. The glycoforms on FN1_N528, ACAN_N333, and N658 showed distinct heterogeneity between OA and control cartilages (summarized in Table 1). The fully sialylated N-glycan (N4H5S2) was exclusively identified on FN1_N258 from control cartilages and mono-sialylated and fucosylated N-glycan (N4H4F1S1) on the same glycopeptides was uniquely detected from OA cartilages. Interestingly, the heterogeneities of glycoforms were observed on ACAN_N333 and N658. The N-glycan of N5H8F1 on ACAN_N333 was only detected in control cartilage, however, five glycoforms (such as N6H4S1 and N4H3F1) on the same site were uniquely detected in OA cartilage. Four complex N-glycans (N5H4F1S2, N6H6F1, N6H7, and N7H6) on ACAN_ N658 were absent in OA cartilage, while the same glycopeptides modified with high mannose N-glycan (N2H11) was significantly increased in OA cartilages compared to controls (*p* < 0.05).

**Table 1:** Heterogeneities of specific glycosylation sites of glycoproteins in OA cartilage

**UniPort Acc**	**Protein name**	**Annotated sequence**	**Glycan composition**	**Foldchange (OA/N)**	***p* value**
**P02751**	Fibronectin (FN1)	DQCIVDDITYNV**N**^528^DTFHK	N4H5S2	0.01	< 0.0001
			N4H4F1S1	100	< 0.0001
**P16112**	Aggrecan core protein (ACAN)	TVYVHA**N**^333^QTGYPDPSSR	N5H8F1	0.01	< 0.0001
			N6H4S1	100	< 0.0001
			N4H3F1	100	< 0.0001
			N5H3F1	100	< 0.0001
			N2H5	100	< 0.0001
			N6H4	100	< 0.0001
		TVYLYP**N**^658^QTGLPDPLSR	N5H4F1S2	0.01	< 0.0001
			N6H6F1	0.01	< 0.0001
			N6H7	0.01	< 0.0001
			N7H6	0.01	< 0.0001
			N2H11	5.98	0.046

*N* HexNAc, *H* Hex, *F* Fuc, *S* NeuAc

### The differential expression of glycosyltransferases in OA cartilages

**Fig. 5:**
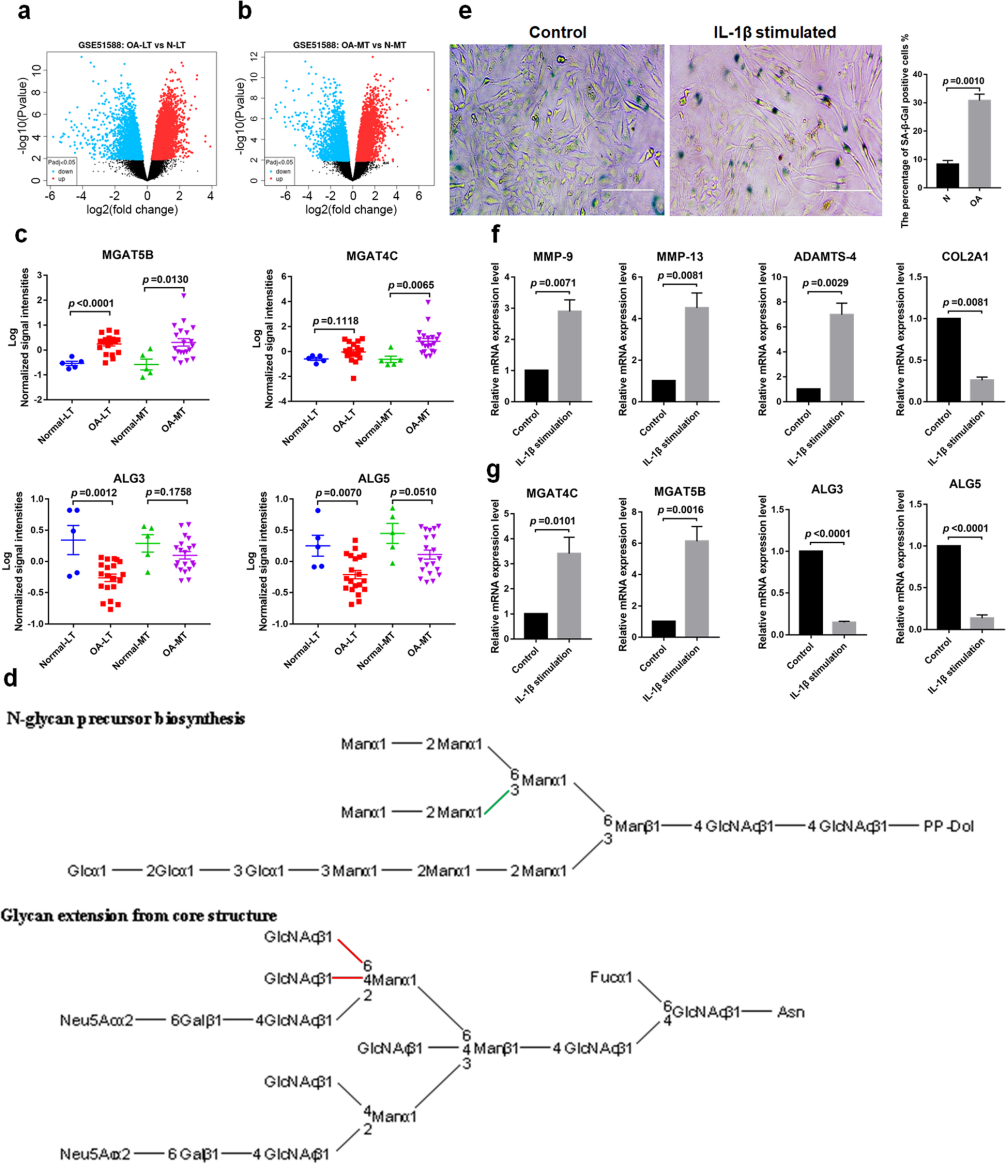
Expression analysis of glycosyltransferases involved in the synthesis of N-glycans. **a**, **b** The differential expression transcripts in OA-LT compared to N-LT (**a**) and OA-MT compared to N-MT (**b**) in GSE51588. **c** The expression levels of MGAT5B, MGAT4C, ALG3, and ALG5 showed evident differences between OA and controls. **d** KEGG pathway map of glycosyltransferases. ALG3 and ALG5 are involved in the synthesis of N-glycan precursor (upper), and MGAT5B and MGAT4C are involved in the synthesis of GlcNAc branching of N-glycans (lower). The linkage catalyzed by ALG3 was marked with a green line, and the linkages catalyzed by MGAT5B and MGAT4C were marked with a red line. **e** The results of SA-β-Gal staining. The C28 cells were treated with or without 10 ng /mL of IL-1β for 48 h and the senescent cells were stained with SA-β-Gal. Quantitative analysis of the percentage of SA-β-Gal positive cells in untreated- versus IL-1β-treated chondrocytes. Data were represented as Mean ± SEM. from three independent experiments. Scale bar = 50 μm. **f** The expression level of MMP-9, MMP-13, ADAMTS-4, and COL2A1 was significantly altered in C28 cells treated with IL-1β. **g** The expression of MGAT4C and MGAT5B was increased and the expression of ALG3 and ALG5 was decreased in C28 cells stimulated by IL-1β

Our results indicated that the high mannose of N-glycans was decreased in OA cartilages. Hence, we assessed the expression of glycosyltransferases involved in the synthesis of N-glycan using the gene expression data set (GSE51588) in the GEO database. There were 9727 and 10,687 transcripts that showed significant differences between OA- knee lateral tibial (LT) plateaus /N-LT and OA- medial tibial (MT) plateaus/N-MT respectively (Fig. 5a, b). Among these, the expression of Mannosyl alpha-1,6-glycoprotein beta-1,6-N-acetyl-glucosaminyltransferase 5B (MGAT5B) and alpha-1,3-mannosylglycoprotein beta-1,4-N-acetylglucosaminyltransferase 4C (MGAT4C) was increased, meanwhile, the expression of alpha-1,3-mannosyltransferase 3 (ALG3) and dolichyl-phosphate beta-glucosyltransferase 5 (ALG5) were decreased in OA-LT and OA-MT compared to controls (Fig. 5c). The ALG3 and ALG5 involved in the synthesis N-glycan precursor, MGAT5B and MGAT4C participated in the synthesis of β1-4/6 GlcNAc branching on complex type of N-glycans (Fig. 5d). Collectively, it suggested the up-regulation of MGATs and down-regulation of ALGs may contribute to the decreased high mannose types of N-glycans in OA cartilages. The OA cell model displayed common characteristics of OA, such as an increased number of senescence cells, elevated expression of MMP-9, MMP-13, and ADAMTS-4, and depressed expression of COL2A1 (Fig. 5e, f). Furthermore, we found the expression of MGATs were increased and ALGs were decreased in cells treated with IL-1β compared to control (Fig. 5g). Our results and GEO data suggested that the pro-inflammatory cytokines not only contribute to the process of OA but also alter the expression of glycosyltransferases and result in aberrant glycosylation in OA cartilage.

## Discussion

Cartilage degradation is not only a characteristic feature but also the main pathology of OA [26]. The ECM of cartilage is remodeled after stimulation by inflammatory cytokines (IL-1β, tissue necrosis factor-α, etc.). Consequently, the alteration of ECM contributes to changes in the biomechanical environment of chondrocytes and promotes the progression of the disease in the presence of inflammation [27]. It is known that the alterations in glycosylation contribute to the process of diseases. A recent study reported that alterations in glycosylation reflected phenotypic changes in osteoarthritic cells [28]. For instance, the aberrant of high mannose-type N-glycans was observed in human and mouse OA cartilage, which is correlated with the release of MMP-13 and ADAMTS-5 in degraded cartilage [29].

Currently, we combined lectin microarray and intact glycopeptide analysis to investigate the alterations in glycosylation associated with OA. Our findings revealed that the high level of α-1,3/6 fucosylation and low level of high-mannose type N-glycan were correlated with OA. Fucosylation is an important modification in cellular glycosylation, which plays important roles in cell adhesion and immune system regulation [30]. Several studies indicated that elevated fucosylation level correlated with inflammatory conditions, and inhibiting fucosylation modulates human nucleus pulposus cell protein translation of catabolic enzymes responses to inflammation [31]. Our previous study elucidated that the high level of fucosylation on TNFR1 facilitated the binding to TNF-α and activated NF-κB and P38/JNK-MAPK pathways in chondrocytes stimulated by TNF-α [32]. High-mannose types of N-glycan are commonly observed in the early stage of the synthesis of N-glycans. These glycans are critical for the correct folding of proteins and protect the carrying proteins from degradation during intracellular transport [33–35]. We found that high mannose types of N-glycan were decreased in OA cartilage, moreover, over 24% of glycopeptides that decreased in OA cartilage were modified with pauci- and high-mannose glycans. Interestingly, heterogeneity in glycosylation was observed among OA samples. Ahmed et al. reported that the glycated, oxidized, and nitrated proteins and amino acids were different between early and advanced OA [36]. On the other hand, the study by Waarsing et al. indicated the existence of distinct subtypes of knee OA with clear differences in structural degradation and symptoms [37]. We supposed that the heterogeneity is associated with the distinct subtypes and degrees of knee OA.

Pro-inflammatory cytokines (such as IL-1β and TNF-α) interact to promote inflammation, elevate the expression of MMP and ADAMTS, and eventually mediate cartilage degradation in OA [38]. The binding of pro-inflammatory cytokines and their receptors activate the RAS-RAF-MEK1/2-ERK1/2 signaling cascade, which promotes the expression of MGAT5B [39–41]. The expression of MGAT4C was also elevated by the stimulation of IL-1β, which is a key pro-inflammatory cytokine in OA [42]. On the other hand, the aberrant expression of glycosyltransferases was also involved in the OA process. The upregulated expression of β-1,2N-acetylglucosaminyltransferase I (GlcNAc-TI) contributed to the synthesis of high-mannose type N-glycans and correlated with overexpression of MMP-13 and ADAMTS-5 [43]. Integrated with these results, we concluded that pro-inflammatory cytokines activate singling pathways (such as NF-κB and RAS-RAF pathways) and promote the expression of FUTs and MGATs and eventually alter the glycoforms of OA cartilages.

The intact glycopeptides were profiled from cartilages to gain insight into the heterogeneity of site-specific N-glycosylation in OA. Overall, 393 intact glycopeptides were identified in cartilage. Among these, 109 N-glycopeptides derived from 47 glycoproteins were exclusive or significantly decreased in OA patients. Particularly, these glycoproteins are mainly located in the extracellular region/space and ECM and are involved in ECM-receptor interaction and complement and coagulation cascade pathways. Our results suggested that the loss of ECM glycoproteins is associated with cartilage degeneration. Interestingly, the heterogeneities of N-glycans on specific glycosylation sites were observed in FN1 and ACAN. It is known that FN1 acts as a bridging molecule in matrix assembly and cell–matrix interfaces. The studies by Luo et al. indicated that the glycopeptide with N-glycosylation at N528 of fibronectin was significantly upregulated in the OA cartilage [44]. The fibronectin fragments promote inflammation and degradation by the induction of cytokine and proteinase expressions, and they also facilitate chondrocyte differentiation via upregulating TGF-β/PI3K/Akt pathways [5, 45, 46]. Our finding revealed heterogeneity of N-glycans on FN1_N528 in OA cartilage may affect fibrin-binding and chondrocyte differentiation. ACAN is a major proteoglycan of articular cartilage. As OA occurred, ACAN was degraded by MMPs and aggrecanases and released sulfated glycosaminoglycan (sGAGs), which is vital for cartilage elasticity via binding to the ACAN core protein to form proteoglycans [47, 48]. We found 6 out of 31 and 5 out of 29 N-glycans on glycosites of N333 and 658 of ACAN showed significant differences between OA and control cartilages. Our findings suggested the heterogeneity of glycans is a new characteristic of OA, and their functional implications still need further investigations.

## Conclusions

In the present study, the abnormal glycopatterns and heterogeneities of site-specific glycosylation associated with OA were investigated by glycomics approaches. Our findings indicated that abnormal glycosylation, including a high level of α-1,3/6 fucosylation and low level of high-mannose type N-glycan, and heterogeneities of glycoforms on FN1_N258, ACAN_N333, and N658 are new features of OA cartilage. Especially, it is the first time to report the heterogeneity of site-specific N-glycans in OA cartilage. Our study provided more information to understand the pathological mechanism of OA.

## Supplementary Information

**Additional file 1. **Supplementary Methods.

**Additional file 2: Table S1.** The clinic information of patients with OA and controls.

**Additional file 3: Table S2.** The information of primers for Real-Time PCR.

**Additional file 4: Table S3.** Altered glycopattern of cartilage proteins between OA and controls based on data of 7 Lectins giving significant differences.

**Additional file 5: Table S4.** Intact glycopeptides identified from OA and control cartilages.

**Additional file 6: Table S5.** The intact glycopeptides that were significantly altered in OA cartilages.

## Data Availability

The data used to support the findings of this study are available from the corresponding author upon request.

## Abbreviations

ACN: Acetonitrile
ALG3: Alpha-1,3-mannosyltransferase 3
ConA: Canavalia ensiformis Agglutinin
DAVID: Database for Annotation, Visualization, and Integrated Discovery
ECM: Extracellular matrix
FA: Formic acid
FDR: False discovery rate
GEO: Gene Expression Omnibus
GlcNAc-TI: β-1,2N-acetylglucosaminyltransferase I
GO: Gene Ontology
H&E: Hematoxylin-eosin
HCA: Hierarchical cluster analysis
KEGG: Kyoto Encyclopedia of Genes and Genomes
LFQ: Label-free quantification
LT: Lateral tibial
MGAT5B: Beta-1,6-N-acetyl-glucosaminyltransferase 5B
MGAT4C: Beta-1,4-N-acetylglucosaminyltransferase 4C
MMPs: Matrix metalloproteinases
MT: Medial tibial
NFIs: Normalized fluorescence intensities
OA: Osteoarthritis
PCA: Principal component analysis
SJA: Sophora Japonica Agglutinin
WGA: *Wheat Germ Agglutinin*
DAPI: 4,6-Diamidino-2-phenylindole
