# Supplementary material for: Characterization of aberrant glycosylation associated with osteoarthritis based on integrated glycomics methods
Source: Arthritis Res Ther. 2023 Jun 12;25:102. doi: 10.1186/s13075-023-03084-w (PMC10258941; doi:10.1186/s13075-023-03084-w)
Supplement: Supplementary file 1 — Additional file 1. Supplementary Methods. [file 13075_2023_3084_MOESM1_ESM.docx]

**Supplementary materials**

**Supplementary Methods**

**Lectin microarray data analysis**

The raw fluorescence intensities of each lectin were obtained from lectin microarray by using GenePix 7.0 software (Axon) set at 70% photomultiplier tube and 100% laser power. The average background was subtracted, and the signal values less than the average background + standard deviations (SD) were excluded for further analysis. To minimize the possible systematic variation, the median of triplicate spots of each lectin for each lectin was globally normalized to the sum of the medians of all the eﬀective data points for each lectin in a block. The normalized medians of each lectin from OA patients (n=13) and controls (n=11) were averaged respectively. Diﬀerences of lectins between OA group and control group were compared based on foldchange (fold changes ≥ 1.5 or ≤ 0.67 and p < 0.05), the significant diﬀerences of lectins between OA patients and controls were evaluated using the Mann-Whitney test using the GraphPad Prism software (Version 7.0, Inc., San Diego, CA).
