# Supplementary material for: Characterization of aberrant glycosylation associated with osteoarthritis based on integrated glycomics methods
Source: Arthritis Res Ther. 2023 Jun 12;25:102. doi: 10.1186/s13075-023-03084-w (PMC10258941; doi:10.1186/s13075-023-03084-w)
Supplement: Supplementary file 2 — Additional file 2: Table S1. The clinic information of patients with OA and controls. [file 13075_2023_3084_MOESM2_ESM.docx]

**Table S1. The clinic information of patients with OA and controls**

|  | OA group (n=13) | Control group (n=11) |
| --- | --- | --- |
| Gender (Male/Female) | 8/5 | 7/4 |
| Age range | 41-68 | 46-57 |
| BMI | 17.2-36.6 | 15.5~ 31.8 |
| Grade of Kellgren-Lawrence | ≥3 | 0 |
