# Supplementary material for: Characterization of aberrant glycosylation associated with osteoarthritis based on integrated glycomics methods
Source: Arthritis Res Ther. 2023 Jun 12;25:102. doi: 10.1186/s13075-023-03084-w (PMC10258941; doi:10.1186/s13075-023-03084-w)
Supplement: Supplementary file 3 — Additional file 3: Table S2. The information of primers for Real-Time PCR. [file 13075_2023_3084_MOESM3_ESM.docx]

**Table S2. The information of primers for Real-Time PCR**

| Symbol Name | Genbank Acc | Primer sequence (5’-3’) | Amplicon size (bp) |
| --- | --- | --- | --- |
| MMP-9 | NM_004994 | Forward: TGTACCGCTATGGTTACACTCG  Reverse: GGCAGGGACAGTTGCTTCT | 97 |
| MMP-13 | NM_002427 | Forward: CCAGACTTCACGATGGCATTG  Reverse: GGCATCTCCTCCATAATTTGGC | 137 |
| ADAMTS‐4 | NM_005099.6 | Forward: ATGGCTCCTATGCCCTCAATG  Reverse: CTGTATCGGAGGCGTGTGTC | 199 |
| COL2A1 | NM_001844 | Forward: CCAGATGACCTTCCTACGCC  Reverse: TTCAGGGCAGTGTACGTGAAC | 186 |
| ALG3 | NM_001006941 | Forward: TTTATGGGGTTGTACTATGCCAC  Reverse: GCAGGTCTGGTGATAGATCAAGA | 102 |
| ALG5 | NM_013338 | Forward: CTAGAGAAGAGACAGAAACGAGAT  Reverse: TGGAAACTTTGTGGCTCCATC | 146 |
| MGAT4C | NM_013244 | Forward: TCACCTATCGCTACCTAGCTG  Reverse: GGCATCACGCCAGGAAGAAT | 200 |
| MGAT5B | NM_001199172 | Forward: ACCCAGTGACCCCAAGTTC  Reverse: CAGGTTGCTTCGGAAAACTGC | 235 |
| GAPDH | NM_001256799 | Forward: CTGGGCTACACTGAGCACC  Reverse: AAGTGGTCGTTGAGGGCAATG | 101 |
