# Supplementary material for: Characterization of aberrant glycosylation associated with osteoarthritis based on integrated glycomics methods
Source: Arthritis Res Ther. 2023 Jun 12;25:102. doi: 10.1186/s13075-023-03084-w (PMC10258941; doi:10.1186/s13075-023-03084-w)
Supplement: Supplementary file 4 — Additional file 4: Table S3. Altered glycopattern of cartilage proteins between OA and controls based on data of 7 Lectins giving significant differences. [file 13075_2023_3084_MOESM4_ESM.docx]

**Table S3. Altered glycopattern of cartilage proteins between OA and controls based on data of 7 lectins giving signiﬁcant differences**

| **Lectin** | **Specificity** | **Normalized fluorescence intensities ^a^** | | **Foldchange**  **(OA/N)** | ***p* value** |
| --- | --- | --- | --- | --- | --- |
|  |  | **Control (n=11)** | **OA (n=13)** |  |  |
| PSA | Fucα-1,6GlcNAc, α-D-Man, α-D-Glc | 0.004 ± 0.003 | 0.017 ± 0.009 | 3.932 | 0.0003 |
| WGA | Multivalent Sia and (GlcNAc)_n_ | 0.027 ± 0.008 | 0.058 ± 0.004 | 2.099 | 0.0107 |
| SJA | Terminal in GalNAc and Gal, anti-A and anti-B human blood group | 0.010 ± 0.004 | 0.019 ± 0.006 | 1.873 | 0.0007 |
| LTL | Fucα1-3Galβ1-4GlcNAc, Fucα1-anti-H blood group specificity | 0.012 ± 0.005 | 0.022 ± 0.005 | 1.835 | 0.0003 |
| HHL | High-Mannose, Manα1-3Man, Manα1-6Man, Man5-GlcNAc2-Asn | 0.056 ± 0.019 | 0.026 ± 0.008 | 0.475 | 0.0020 |
| UEA-I | Fucα1-2Galβ1-4Glc(NAc) | 0.019 ± 0.010 | 0.010 ± 0.006 | 0.534 | 0.0187 |
| ConA | High-Mannose, Manα1-6(Manα1-3)Man, αMannose, αGlc | 0.079 ± 0.038 | 0.042 ± 0.012 | 0.535 | 0.073 |

^a^ The normalized fluorescence intensities were represented as Mean with SEM.
